# European Guideline (EuroGuiDerm) on atopic eczema: Living update

**DOI:** 10.1111/jdv.20639

**Published:** 2025-05-02

**Authors:** A. Wollenberg, M. Kinberger, B. Arents, N. Aszodi, S. Barbarot, T. Bieber, H. A. Brough, P. Calzavara‐Pinton, S. Christen‐Zaech, M. Deleuran, M. Dittmann, N. Fosse, K. Gáspár, L. A. A. Gerbens, U. Gieler, G. Girolomoni, S. Gregoriou, S. Holland, C. G. Mortz, A. Nast, U. Nygaard, E. M. Rehbinder, J. Ring, M. Rossi, E. Serra‐Baldrich, D. Simon, Z. Z. Szalai, J. C. Szepietowski, A. Torrelo, T. Werfel, R. N. Werner, C. Flohr

**Affiliations:** ^1^ Department of Dermatology and Allergology University Hospital Augsburg Augsburg Germany; ^2^ Department of Dermatology and Allergy Ludwig Maximilian University Munich Munich Germany; ^3^ Comprehensive Center for Inflammatory Medicine University of Luebeck Luebeck Germany; ^4^ Division of Evidence‐Based Medicine (dEBM), Department of Dermatology, Venereology and Allergology Charité – Universitätsmedizin Berlin, Corporate Member of Freie Universität Berlin and Humboldt‐Universität Zu Berlin Berlin Germany; ^5^ European Federation of Allergy and Airways Diseases Patients' Associations (EFA) Brussels Belgium; ^6^ Department of Dermatology Nantes Université, CHU Nantes, UMR 1280 PhAN, INRAE Nantes France; ^7^ Christine Kühne‐Center for Allergy Research and Education, Medicine Campus Davos Switzerland; ^8^ Children's Allergy Service, Evelina London Children's Hospital Guy's and St. Thomas' NHS Foundation Trust London UK; ^9^ Paediatric Allergy Group, Department of Women and Children's Health, School of Life Course Sciences King's College London London UK; ^10^ Dermatology Department University of Brescia Brescia Italy; ^11^ University Hospital Lausanne Lausanne Switzerland; ^12^ Department of Dermatology Aarhus University Hospital and Aarhus University Aarhus Denmark; ^13^ Department of Dermatology University Hospital Basel Basel Switzerland; ^14^ Department of Dermatology, Faculty of Medicine University of Debrecen Debrecen Hungary; ^15^ Department of Dermatology, Amsterdam UMC, Academic Medical Center, Amsterdam Public Health & Infection and Immunity University of Amsterdam Amsterdam The Netherlands; ^16^ Department of Dermatology University of Giessen Giessen Germany; ^17^ Dermatology and Venereology Section, Department of Medicine University of Verona Verona Italy; ^18^ Faculty of Medicine National and Kapodistrian University of Athens Athens Greece; ^19^ Eczema Outreach Support (UK) Linlithgow UK; ^20^ Department of Dermatology and Allergy Centre, Odense University Hospital University of Southern Denmark Odense Denmark; ^21^ Department of Dermato‐Venerology Aarhus University Hospital Aarhus Denmark; ^22^ Dermatology Department Oslo University Hospital Oslo Norway; ^23^ Clinical Medicine University of Oslo Oslo Norway; ^24^ Department of Dermatology and Allergology Biederstein Technical University Munich Munich Germany; ^25^ Dermatology Hospital of Sant Pau Barcelona Spain; ^26^ Department of Dermatology, Inselspital, Bern University Hospital University of Bern Bern Switzerland; ^27^ Pediatric Dermatology Unit Heim Pál National Children's Institute Budapest Budapest Hungary; ^28^ Department of Dermato‐Venereology 4th Military Hospital Wroclaw Poland; ^29^ Faculty of Medicine Wroclaw University of Science and Technology Wroclaw Poland; ^30^ Hospital Infantil Niño Jesús Madrid Spain; ^31^ Department of Dermatology and Allergy Hannover Medical School Hannover Germany; ^32^ St John's Institute of Dermatology King's College London London UK; ^33^ Guy's & St Thomas' NHS Foundation Trust London UK

## Abstract

The evidence‐ and consensus‐based living guideline on atopic eczema was developed in accordance with the EuroGuiDerm Guideline and Consensus Statement Development Manual. The original EuroGuiDerm Guideline on atopic eczema was published in June 2022. Since then, the part of the guideline dealing with systemic therapy has been updated twice. This paper summarizes the results of the second update. Twenty‐eight experts (including clinicians and patient representatives) from 12 European countries participated. The updated guideline provides guidance on which patients should be treated with systemic therapies, as well as recommendations and detailed information on each systemic drug. The systemic treatment options discussed in the guideline comprise conventional immunosuppressive drugs (azathioprine, ciclosporin, glucocorticosteroids, methotrexate and mycophenolate mofetil), biologics (dupilumab, lebrikizumab, nemolizumab and tralokinumab) and Janus kinase (JAK) inhibitors (abrocitinib, baricitinib and upadacitinib). Additionally, the updated guidelines address considerations for paediatric, adolescent, pregnant and breastfeeding patients. For all other aspects, please refer to the 2022 version.

## Graphical abstract

The updated guideline includes a new evidence‐based recommendation for the IL‐13 inhibitor lebrikizumab. In addition, the Janus kinase inhibitors baricitinib and abrocitinib, which were initially only approved for adult patients, are now also recommended for children from the age of 2 and adolescents from the age of 12, respectively.

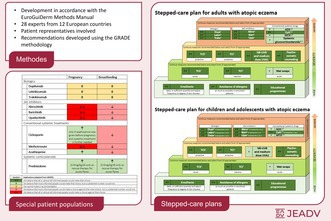

**Why was the study undertaken?**

- This living guideline update provides up‐to‐date guidance on systemic therapies for atopic eczema.

**What does this study add?**

- The newly approved interleukin 13 inhibitor lebrikizumab has been incorporated into our recommendations. In addition, the Janus kinase inhibitors baricitinib and abrocitinib, which were initially only approved for adult patients, are now also recommended for children from the age of 2 and adolescents from the age of 12, respectively.

**What are the implications of this study for disease understanding and/or clinical care?**

- With three approved and recommended biologics (dupilumab, lebrikizumab and tralokinumab) and three Janus kinase inhibitors (abrocitinib, baricitinib and upadacitinib), the range of advanced systemic therapies for the treatment of atopic eczema continues to expand, not just for adults but also for paediatric patients.

## METHODS

For detailed information on the methodology of the guideline development and the development of the update of the guideline, see the methods report.

The wording of the recommendations was standardized (as suggested by the GRADE Working Group^1^); see Table 1.

**TABLE 1:** Wording of recommendations.

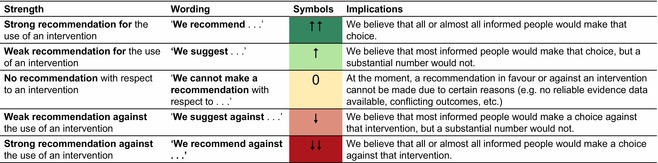

This second update of the guideline was initiated to include lebrikizumab. Lebrikizumab was approved by the EMA in November 2023, necessitating a formal update. In addition, baricitinib and abrocitinib received an extension of approval for use in children and adolescents from 2 years of age and older and for use in adolescents from 12 years of age and older, respectively. Furthermore, an update of the network meta‐analysis by Drucker et al.^2^ was published in July 2024.

The new evidence from the updated network meta‐analysis and the changes prepared by the coordinators and the EuroGuiDerm team in the background text of the chapters on systemic treatment and new versions of the stepped‐care plans and drug tables were presented to the guideline development group (GDG) and voted on in an online survey. In addition, a new recommendation on lebrikizumab and two modified recommendations from the sections on pregnancy and breastfeeding in which lebrikizumab was added were voted on.

All experts were asked to vote (agree, abstain, reject). Alternative suggestions could be entered as a reply option. Voting was anonymous. Only the EuroGuiDerm team had access to the results. All authors could participate in the recommendation voting, but the votes of those with personal financial conflicts of interest did not count.

For this update, the group comprised 28 experts from 12 countries. Ten experts (35.7%) declared personal‐financial conflicts of interest.

### Evidence

The living systematic review by Drucker et al.^2^, ^3^ was used as the evidence base on which we created an evidence‐to‐decision framework (see evidence report). Challenges exist with comparing clinical trials in atopic eczema (AE) due to their differences in trial design, including study comparators, rules for rescue treatment, washout periods for topical and systemic treatments, inclusion criteria and the duration of the screening period.^4^ The analysis by Drucker et al.^2^, ^3^ does not take into consideration the overall management plan that targets long‐term stabilization, flare prevention and avoidance of side effects beyond 16 weeks.^5^ We only summarize the results here. For further information, please refer to^2^, ^3^ https://eczematherapies.com.

For each recommendation that is evidence‐based, we added the certainty of the evidence compared to placebo.^2^, ^3^ The assessment of the certainty of evidence leads to four grades; see Evidence‐box above (tab. 5.1. GRADE Handbook^6^).

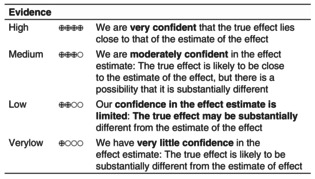

## OVERVIEW OF RECOMMENDATIONS

**FIGURE 1:**
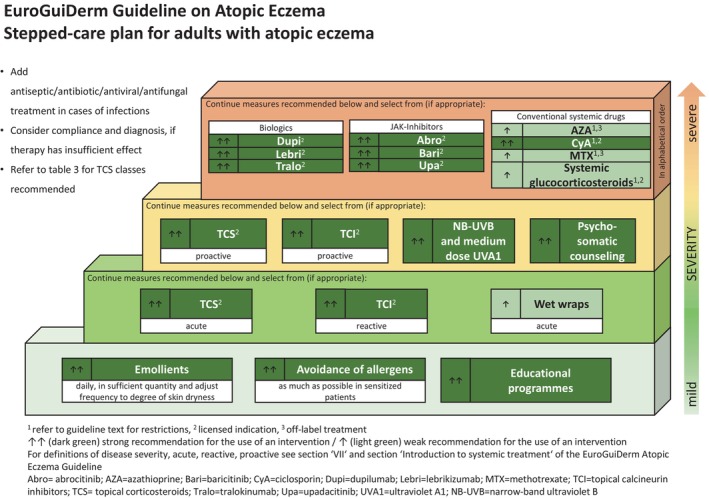
Stepped‐care plan for adults with atopic eczema.

**FIGURE 2:**
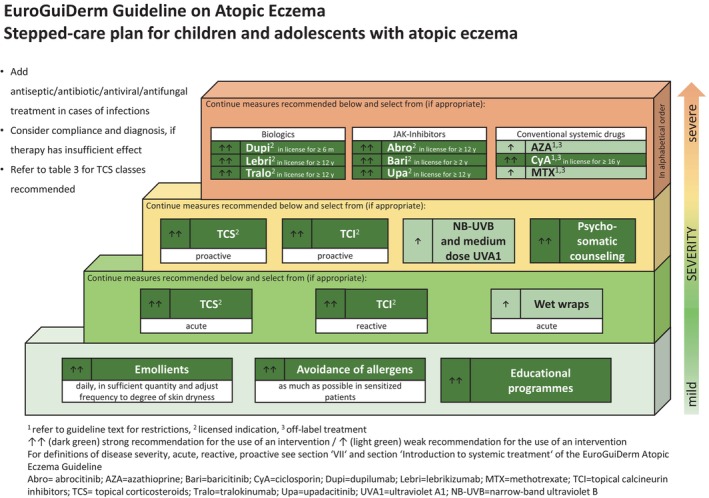
Stepped‐care plan for children and adolescents with atopic eczema.

Below you find the updated stepped‐care plans for the treatment of AE in adults (Figure 1) and in children and adolescents (Figure 2).

Table 2 shows general recommendations for systemic drugs for adult AE patients who are candidates for systemic treatment.

**TABLE 2:** General recommendations for systemic drugs for AE adult patients who are candidates for systemic treatment (for details see corresponding chapter).

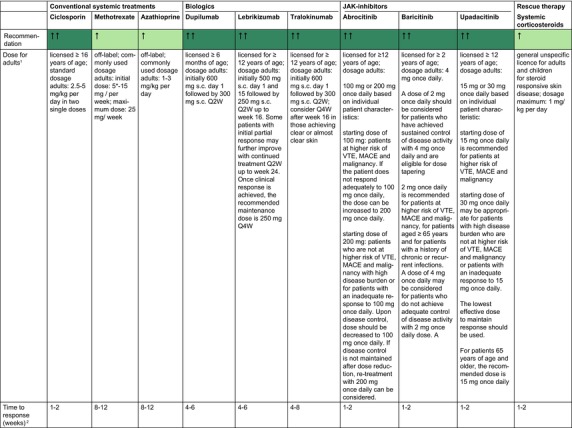
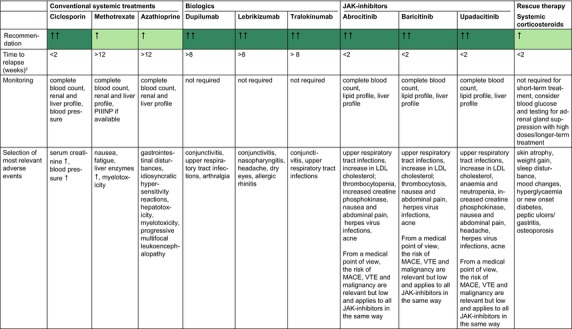

Abbreviations: ↑, rise; AE, atopic eczema; GL, guideline; LDL, low‐density lipoprotein; MACE, major adverse cardiovascular events; PIIINP, procollagen III N‐terminal propeptide; TPMT, thiopurine‐S‐methyltransferase; VTE, venous thromboembolism.

^a^ SmPC.

^b^ Expert experience. *5 mg can be administered as a test dose.

Table 3 shows general recommendations for systemic drugs for special atopic eczema patient populations.

**TABLE 3:** General recommendations for systemic drugs for special AE patient populations (for details see corresponding chapter).

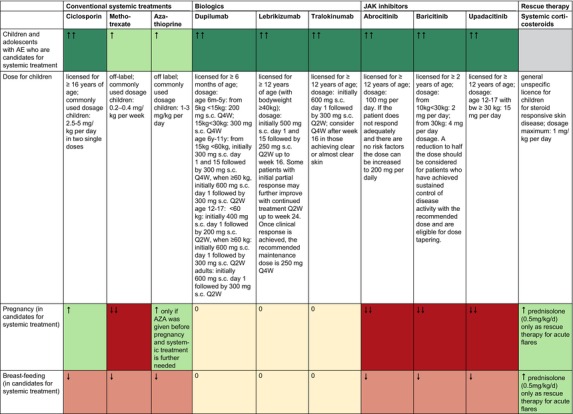

Abbreviations: bw, bodyweight; Q2W, once every 2 weeks; Q4W, once every 4 weeks.

^a^ SmPC.

## INTRODUCTION TO SYSTEMIC TREATMENT

The area of systemic therapy of AE has flourished during the last few years, as many new substances are marketed, licensed or in the last step of clinical development. The licensing programmes of the various new biologics and small molecules are providing much better levels of evidence than those available for the longer existing drugs due to more robust RCT evidence.

Systemic therapy of AE is deemed necessary if the signs and symptoms of AE cannot be controlled sufficiently with appropriate topical treatments and UV‐light therapy. Systemic therapy can also be useful to reduce the total amount of topical corticosteroids (TCS) in patients who need large amounts of potent TCS over prolonged periods to control their AE.

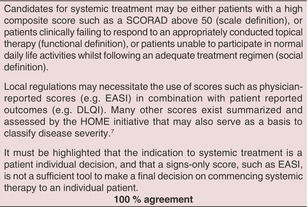

Before starting systemic treatment, it is important to rule out relevant differential diagnoses such as cutaneous T‐cell lymphoma and, in selected cases, primary immunodeficiency syndromes,^8^ and to ascertain that potential trigger factors such as allergic contact dermatitis have been excluded, and behavioural as well as educational reasons for poor responses have been adequately addressed.

Conventional systemic immunosuppressants, such as systemic corticosteroids (SCS), ciclosporin, azathioprine (AZA), mycophenolate mofetil (MMF), enteric‐coated mycophenolate sodium (EC‐MPS) and methotrexate (MTX) were used traditionally for difficult‐to‐treat AE. Most were not licensed for this indication. These drugs may roughly be divided into two groups: SCS and ciclosporin have a rapid onset of action and can be used to treat flares of AE or to bridge the time until the onset of action of slower‐acting systemic immunosuppressants such as MTX, AZA and MMF/EC‐MPS. The kinetics of the novel Janus kinase inhibitors baricitinib (Bari), abrocitinib (Abro) and upadacitinib (Upa) place these agents in the fast‐acting group, whereas the Th2‐blocking agents (like IL13 or IL13/IL4 inhibitors) dupilumab (Dupi), tralokinumab (Tralo) and lebrikizumab (Lebri), as well as the IL31‐receptor blocking agent nemolizumab (Nemo) need some weeks to reach full efficacy.

The following recommendations for systemic drugs are based on the living systematic review by Drucker et al.,^2^, ^3^ other published literature and medical considerations, as well as expert opinion, and may differ from the legal licensing status and access routes, which are not uniform in European countries.

## CONVENTIONAL SYSTEMIC DRUGS

### Azathioprine

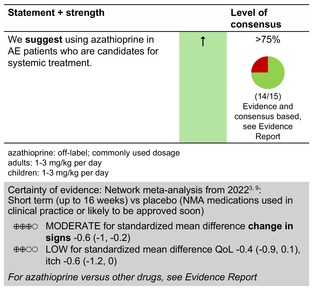

#### Mechanisms of action and efficacy

AZA is a pro‐drug that is rapidly converted in vivo to the anti‐metabolite 6‐mercaptopurine (6‐MP), following cleavage of its imidazole side chain. It is believed to exert its primary immunosuppressant effect via metabolites of 6‐MP, thioguanine nucleotides (TGNs), which are subsequently incorporated into DNA, inhibiting its synthesis.^10^

The efficacy of AZA is comparable to that of MTX but lower compared to dupilumab and ciclosporin in clearing clinical signs of AE.^11^

Randomized clinical trials report a significant superiority of AZA versus placebo, with a decrease in clinical scores such as Six Area, Six Sign Atopic Dermatitis (SASSAD) and Scoring Atopic Dermatitis by 26%–39% after 12 weeks.^12^ However, results from retrospective studies are less favourable, with a percentage of AZA treatment failure varying from 30% to 57% due to adverse effects or lack of effectiveness.^13^, ^14^, ^15^ An observational follow‐up study of 36 adult patients with severe AE treated with MTX or AZA over a 24‐week period demonstrated less improvement in subjects with filaggrin mutations (36%, 13/36) compared to those without filaggrin mutations.^12^

Long‐term studies on adult patients treated with either AZA or MTX showed a relative reduction in SCORAD of 53% (*p* < 0.01) and 63% (*p* < 0.01) after 2 years, and 54% and 53% after 5 years, respectively.^12^, ^16^ Patients with a Filaggrin mutation seemed to have slower but prolonged effects of therapy compared with patients without a mutation.^12^, ^16^

#### Dosage: acute flare, short term, long term

- Off‐licence
- Commonly used dosage
  - Adults and children: 1–3 mg/kg bodyweight per day
  - If no improvement of AE occurs within 3 months, withdrawing azathioprine should be considered.
- We recommend combining AZA, as any systemic treatment, with emollients and, whenever needed, topical anti‐inflammatory treatment in AE patients.
- If timely thiopurine S‐methyltransferase (TPMT) activity measurement is available, the following dosing of AZA has been suggested:
  - Very low activity (<2.5 per mL red blood cells [RBC]), treatment should not be started
  - Intermediate activity (2.5–7.5 nmol/h/mL RBC): 0.5 mg/kg body weight per day for the first 4 weeks and then increase to 1.0 mg/kg body weight per day
  - Normal activity (>7.5 nmol/h/mL RBC): 2.0 mg/kg bodyweight per day for the first 4 weeks and then increase to 2.5–3.0 mg/kg bodyweight per day

Low azathioprine doses (0.5–1.0 mg/kg bodyweight per day) for the first 4 weeks were shown to reduce gastrointestinal side effects.^17^

If TPMT results are not available prior to starting AZA therapy, then half the standard treatment should be given for about 4–6 weeks under close monitoring of full blood count and liver profile, prior to going up to the full treatment dose.

#### Safety

In the short and medium term, the most commonly reported serious dose‐dependent effects are hepatotoxicity and myelotoxicity, together with gastrointestinal disturbances. Further, idiosyncratic hypersensitivity reactions (e.g. fever, rigours, myalgia, arthralgia and occasionally pancreatitis) may occur.^18^

Concerns have been raised about the potential carcinogenicity induced by long‐term treatment with azathioprine (predominantly squamous cell skin cancer and non‐Hodgkin's lymphoma), especially if AZA is combined with other immunosuppressant regimens.^19^

#### Monitoring

- Baseline: Complete blood count, renal and liver profile.
- TPMT activity if available.
- Screening for chronic infections (e.g. hepatitis B‐/C, HIV) before therapy should be considered.
- Follow‐up: Complete blood count, renal and liver profile twice monthly for 2 months, monthly for 4 months, then every 2–3 months and with dose increases.
- Pregnancy testing before and during AZA therapy where indicated.

#### Combination with other treatments

Concomitantly to AZA, topical therapy with corticosteroids and/or calcineurin inhibitors can be applied.

Because of a potentially increased risk to develop skin cancer, AZA should not be combined with UV light (UVA, UVB, PUVA).

#### Special considerations

There is a theoretical risk of teratogenesis with AZA. This is based on studies in animals in which very high doses of AZA were used. However, in practice, AZA has been used for over 30 years in sexually active men and women, and no definite association between the drug and the incidence of fetal abnormalities has been observed. There also seems to be no effect on fertility.

According to a position paper by ETFAD,^20^ AZA use during pregnancy should be avoided as there are better options, but may be used off‐label in the absence of other alternatives as continuation of treatment in women already receiving this treatment at the time of conception. According to experts' opinion of ETFAD, the dosage of azathioprine should be reduced by 50% if it is continued during pregnancy. Initiation of azathioprine after conception is not recommended.

The use of AZA during lactation is debated. The WHO has recommended that the potential side effects of AZA outweigh the effects and benefits of the treatment,^21^ and studies suggest that AZA intake during breastfeeding could increase the long‐term risk of immunosuppression and carcinogenesis in the child.^22^

AZA is not licensed for the treatment of AE in children, but it has proven beneficial in several retrospective paediatric case series. The main disadvantage of AZA is that it reaches its maximum treatment effect only after 3–4 months.^23^

### Ciclosporin

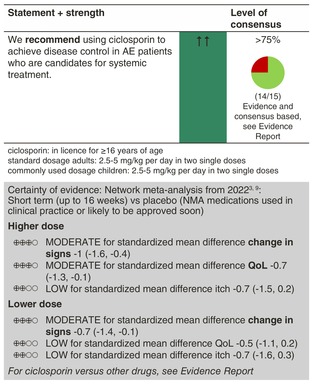

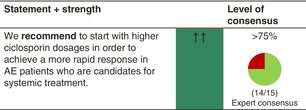

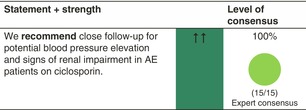

#### Mechanisms of action and efficacy

Ciclosporin inhibits T‐cell activation and proliferation by blocking nuclear factor of activated T cell (NFAT)‐dependent cytokine production.

Ciclosporin has been approved for the treatment of AE in adults in many European countries and is considered a first‐line option for patients with severe disease if other, novel therapies are not available or indicated. Ciclosporin is very effective for AE in both children and adults, with a better tolerability in children.^24^, ^25^, ^26^ Although similarly effective in the NMA by Drucker et al. evaluating adult trials up to 16 weeks and in a real‐world analysis from the UK‐Irish Atopic Eczema Systemic TherApy Register (A‐STAR) among both adults and children,^27^ real‐life data reveal a longer drug survival of dupilumab compared to ciclosporin after 16 months, but similar effectiveness.^11^, ^28^ In head‐to‐head adult trials, ciclosporin was superior to MTX, prednisolone, IVIG, UVA and UVB, and similarly efficacious as enteric‐coated mycophenolate sodium (EC‐MPS).^12^, ^29^

In an investigator‐blinded randomized controlled trial comparing ciclosporin (4 mg/kg/day) with MTX (0.4 mg/kg/week) in children aged 2–16 years (*n* = 103), participants receiving ciclosporin showed a faster response to treatment by 12 weeks (TREAT trial). However, MTX was superior after this time point, with more sustained disease control seen up to Week 60, even after treatment was stopped at 36 weeks (lower disease severity measured by EASI and o‐SCORAD as well as less patient‐reported flares and reduced need for the use of topical anti‐inflammatory treatment), suggesting potential disease modification through MTX.^30^ Both drugs improved QoL above the minimal important difference for the CDLQI and were adequately tolerated and there were no serious adverse events attributed to either medication. In addition, there was no significant impact of either treatment on renal tubular function, using sensitive tubular biomarkers.^30^ In the short‐term treatment of AE, higher ciclosporin dosages (5 mg/kg/day) lead to a more rapid response and are more efficacious than lower dosages (2.5–3 mg/kg/day).^12^ Long‐term use of ciclosporin up to 1 year can be recommended based on several trials; however, their evidence is limited because of the open‐label design and high dropout rates.^12^

#### Dosage: Acute flare, short term, long term

- Licensed ≥16 years of age
- Standard dosage adults: 2.5–5 mg/kg body weight per day in two single doses
  - Acute flare, short‐term: 4–5 mg/kg body weight per day
  - Long‐term: 2.5–3 mg/kg body weight per day
- Commonly used dosage children: 2.5–5 mg/kg body weight per day in two single doses
- We recommend combining ciclosporin, as any systemic treatment, with emollients and, whenever needed, topical anti‐inflammatory treatment in AE patients

#### Safety

Ciclosporin has a narrow therapeutic index and requires a close follow‐up for blood pressure and signs of renal impairment. To note, clinically relevant increases of creatinine seem less common than expected, as recently confirmed in the TREAT trial, which showed no abnormal renal profiles due to ciclosporin, even when sensitive tubular function biomarkers were used (see above).^15^, ^25^, ^30^

#### Monitoring

- Blood pressure, full blood count, renal and liver profile (including GGT) according to national guidelines (e.g. at baseline, 4 weeks and then 3‐monthly).
- Screening for hepatitis B/C and HIV before therapy should be considered.

#### Combination with other treatments

Concomitantly with ciclosporin, topical therapy with corticosteroids and/or calcineurin inhibitors can be applied.

Because of a potentially increased risk to develop skin cancer, ciclosporin should not be combined with UV light (UVA, UVB, PUVA).

#### Special considerations

Ciclosporin has been shown to be effective, safe and well tolerated in children and adolescents.^24^, ^26^, ^31^

Ciclosporin can be considered in pregnant women with severe AE. So far, no increased risk of congenital malformations or fetal death compared to the background populations has been reported. An increased risk of low birthweight cannot be ruled out.^20^ Where systemic therapy is likely to be needed throughout pregnancy, ciclosporin is the first choice therapy.^20^

### Systemic glucocorticosteroids

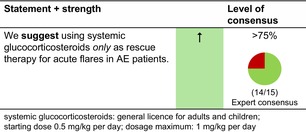

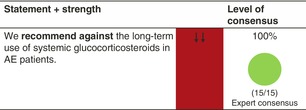

#### Mechanisms of action and efficacy

Glucocorticoids are a class of steroid hormones that bind to the glucocorticoid receptor. The activated glucocorticoid receptor complex upregulates the expression of anti‐inflammatory proteins and suppresses the expression of pro‐inflammatory proteins, leading to a broad anti‐inflammatory property.^32^

There are only a few studies in adult and paediatric AE patients, despite the regular use of systemic glucocorticosteroids in clinical practice. In studies conducted on children and adults, systemic glucocorticosteroids do not induce long‐term remission and swift rebound is common. Systemic glucocorticosteroids have significantly inferior efficacy to ciclosporin.^24^, ^33^

#### Dosage: Acute flare, short term, long term

- Acute flare: Starting dose is usually 0.5 mg/kg body weight per day. Treatment should be discontinued or tapered as soon as possible.
- Short term and long term: no relevant dosing
- We recommend combining systemic glucocorticosteroids, as any systemic treatment, with emollients and, whenever needed, topical anti‐inflammatory treatments in AE patients.

#### Safety

Systemic glucocorticosteroids have a wide therapeutic index. Toxicity is related to the mean dose, cumulative dose and duration of use. At high doses and with long‐term use (typically >0.5 mg/kg/day) important side effects include skin atrophy, weight gain, sleep disturbance, mood changes, hyperglycaemia or new‐onset diabetes, peptic ulcers/gastritis, osteoporosis and increased susceptibility to infections.^34^ In particular, with long‐term use, patients can also develop adrenal suppression and together with a high risk of rebound flares when tapering the treatment dose, cessation can be challenging. Systemic glucocorticosteroids must therefore be avoided as a long‐term treatment in adults and children. Even a fairly high dose can simply be stopped without tapering when used for no longer than 3 weeks.^35^

#### Monitoring

No standard set of variables is recommended when used for acute rescue therapy, but patients' individual needs for monitoring may apply.

#### Combination with other treatments

There are none of the other treatments in AE that are contraindicated when using systemic glucocorticosteroids.

#### Special considerations

Treatment of acute flares of AE with oral glucocorticosteroids is moderately effective.^24^, ^33^

Systemic glucocorticosteroids have an unfavourable risk/benefit ratio for the long‐term treatment of adult and paediatric AE.

### Methotrexate

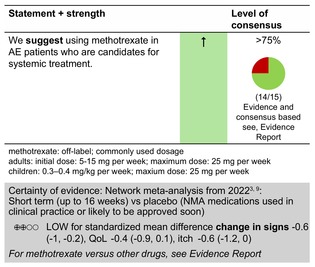

#### Mechanisms of action and efficacy

MTX is a folic acid antagonist that impedes cell division, DNA/RNA synthesis and repair and protein synthesis, altogether suppressing the activity of the immune system. Although its exact action in AE is not fully understood, inhibition of the JAK/STAT pathway has been proposed.^36^

MTX has been used in the treatment of moderate and severe AE for years, but clinical trial evidence is limited. Consequently, recommendations have been primarily based on expert consensus,^37^, ^38^, ^39^, ^40^ but there is one controlled study comparing MTX with AZA in adults,^16^, ^41^, ^42^ one investigator‐blinded randomized controlled trial comparing MTX with ciclosporin in children (TREAT trial)^26^ and an open‐label randomized multi‐centre study in children, again comparing MTX with ciclosporin.^43^ Altogether, these studies support that MTX can be considered effective, relatively safe and well‐tolerated treatments for severe AE both in children and adults—findings also in keeping with recent retrospective studies.^44^, ^45^, ^46^

In the TREAT trial (*n* = 103) ciclosporin showed a faster response by 12 weeks (4 mg/kg/day), but MTX (0.4 mg/kg/week) was superior after this time point, with more sustained disease control seen up to Week 60, even after treatment was stopped at 36 weeks (lower disease severity measured by EASI and o‐SCORAD, as well as less patient‐reported flares and reduced need for the use of topical anti‐inflammatory treatment), suggesting potential disease modification through MTX. Both MTX and ciclosporin improved QoL above the minimally important difference for the CDLQI. Importantly, ciclosporin was significantly more expensive than MTX (£0.55 per week) versus ciclosporin (£24.33 per week, UK National Health Service prices 2023), which should be taken into account when treatment with either drug is considered.^26^

In the living network meta‐analysis by Drucker et al., which only included adults, the efficacy of MTX was comparable to AZA and lower than dupilumab and ciclosporin in clearing clinical signs of AE at eek 16. In addition, the UK‐Irish Atopic Eczema Systemic TherApy register (A‐STAR) recently published a real‐world comparison of MTX versus ciclosporin and dupilumab.^11^, ^27^ Ciclosporin and dupilumab were superior to MTX with regard to treatment effectiveness, while all three medications had a similar impact on QoL. It is important to keep in mind that gradual up‐dosing was commonly used in those receiving MTX in the A‐STAR analysis, while the TREAT trial regimen involved patients receiving the full treatment dose from the outset.^37^, ^38^, ^39^ One adult study suggests that patients who do not benefit from a moderate weekly dose (10–15 mg) of MTX over a 3‐month treatment period will probably not benefit from an increased dosage. However, slow gradual up‐dosing of MTX might underestimate the therapeutic potential of the drug in AE. In children, 0.4 mg/kg/week is recommended, which is significantly higher than dosing in adults.^37^ 25 mg/week is the widely used maximum treatment dose for adult and paediatric AE patients.^40^

#### Dosage: Acute flare, short term, long term

- Off licence
- Commonly used dosage
  - Adults: initial dose: 5*–15 mg/week; maximum dose: 25 mg/week (*5 mg can be administered as a test dose)
  - Children: 0.2–0.4 mg/kg/week
- Oral and subcutaneous delivery are considered equivalent options for administration. For patients in whom MTX 15–25 mg orally once weekly is ineffective or poorly tolerated, a trial of subcutaneous MTX administration is an alternative.
- We recommend combining MTX, as any systemic treatment, with emollients and, whenever needed, topical anti‐inflammatory treatment in AE patients.
- Concomitant use of folic acid should be considered to reduce gastrointestinal and other side effects related to the folic acid antagonistic effect of the drug.^47^

#### Safety

As MTX is a commonly used drug in dermatology, the safety profile is well recognized, with nausea, fatigue and raised liver enzymes as main side effects, while pancytopenia and idiopathic pulmonary fibrosis are of key concern but only very rarely seen.

MTX is generally well tolerated and is considered safe for long‐term treatment, based on experience and multiple studies including both adults and children suffering from psoriasis and rheumatologic disease.^48^, ^49^

#### Monitoring

Complete blood count, renal and liver profile before and every 4 weeks for the first 3 months, or after increasing the dose, then every 8–12 weeks.

In patients undergoing long‐term MTX therapy, a fibroscan may be considered an option to monitor liver fibrosis in accordance with national and localguidelines. An emerging alternative is the fibrosis 4 index, combining age, AST, ALT and platelet count to identify those at increased risk of liver fibrosis.^50^

Screening for chronic infections (e.g. hepatitis B‐/C, HIV, tuberculosis) before therapy should be considered.

Any noteworthy impact on liver or bone marrow function should give cause for dose reduction or transient or total discontinuation of treatment.

#### Combination with other treatments

Combination with TCS, TCI or narrow band UVB phototherapy is an established treatment combination and considered safe. Concomitant use of ciclosporin is a relative contraindication. There is experience from rheumatoid arthritis for combining with the JAK inhibitor baricitinib.

#### Special considerations

MTX may be used for the treatment of AE in both adults and children.

Subcutaneous administration increases bioavailability and tolerability, as well as adherence compared to oral treatment.

MTX affects fertility and is teratogenic. Fertile women should use effective contraception. The same is recommended for men treated with MTX who are living with a woman of childbearing potential.

### Mycophenolate mofetil

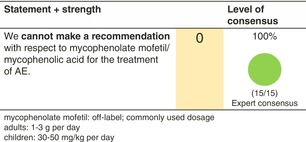

#### Mechanisms of action and efficacy

Mycophenolate mofetil is a prodrug of mycophenolic acid (MPA), an inhibitor of inosine‐5v‐monophosphate dehydrogenase. MPA depletes guanosine nucleotides preferentially in T and B lymphocytes and inhibits their proliferation. MPA also inhibits the glycosylation and expression of adhesion molecules, as well as the recruitment of lymphocytes and monocytes into sites of inflammation.^51^

A recent systematic review and meta‐analysis^52^ including 18 studies with a total of 140 adult and paediatric patients evaluated the efficacy of off‐label use of MMF in patients with AE refractory or not tolerating other first‐line systemic agents. There was a significant reduction in pre‐ to post‐SCORAD scores by 18 points (*p* = 0.0002) with 77.6% of patients reporting partial or full remission. Relapses occurred in 8.2% of cases. The average time for initial effects was 6.8 ± 7 weeks.

#### Dosage: Acute flare, short term, long term

- Off licence
- Commonly used dosage
  - Adults: 1–3 g/day
  - Children: 30–50 mg/kg bodyweight per day
  - Typically given in two divided doses
- For patients receiving systemic treatment with MMF, we recommend additional therapy with emollients and, if necessary, topical anti‐inflammatory therapy.

#### Safety

- The most common side effects include headaches and gastrointestinal symptoms, followed by infections, especially during long‐term therapy.
- Haematological adverse effects include anaemia, leukopenia, neutropenia and thrombocytopenia, albeit rarely.

#### Monitoring

- Complete blood count, renal and liver profile before therapy, then every 2 weeks for 1 month; monthly for 3 months; and every 2–3 months thereafter.
- Screening for chronic infections (e.g. hepatitis B‐/C, HIV) according to national and local guidelines.
- Pregnancy testing before and during MMF therapy if indicated.

#### Combination with other treatments

Concomitantly to MMF, topical therapy with corticosteroids and/or calcineurin inhibitors can be applied.

#### Special considerations

In case series, the efficacy and safety of MMF in children has been investigated.

## BIOLOGICS

### Dupilumab

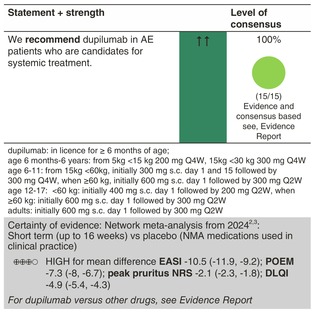

#### Mechanisms of action and efficacy

Dupilumab is the first marketed fully human IgG4 monoclonal antibody (mAb) in the treatment of AE and has been available for the treatment of adults for more than 6 years in many countries. It is now also approved for children aged 6 months and over. Dupilumab binds to the α‐subunit of the IL‐4 receptor, which is part of both the IL‐4 and IL‐13 receptor complex. The safety and efficacy of dupilumab were primarily established in placebo‐controlled studies in moderate‐to‐severe AE.^53^ Dupilumab showed significant clinical effects across three distinct severity assessment tools: Eczema Area and Severity Index (EASI), Investigator's Global Assessment (IGA) and SCORing Atopic Dermatitis (SCORAD). Moreover, dupilumab treatment significantly reduced pruritus. Dupilumab has shown efficacy in both intrinsic and extrinsic AE.^54^ Dupilumab is also registered for the treatment of moderate‐to‐severe asthma, eosinophilic esophagitis and chronic rhinosinusitis with nasal polyps, thereby covering several Type 2 inflammatory diseases.

#### Dosage: Acute flare, short term, long term

The approved dosing of dupilumab in adults consists of a 600‐mg subcutaneous loading dose followed by maintenance doses of 300 mg every other week (Q2W). For children, the following dosing regimens are used: licensed for ≥6 months; age 6 months to 6 years: from 5 kg <15 kg 200 mg Q4W, 15 kg <30 kg 300 mg Q4W. Age 6–11: from 15 kg <60 kg, initially 300 mg s.c. Days 1 and 15 followed by 300 mg Q4W; when ≥60 kg, initially 600 mg s.c. Day 1 followed by 300 mg Q2W. Age 12–17: <60 kg: initially 400 mg s.c. Day 1 followed by 200 mg Q2W; when ≥60 kg: initially 600 mg s.c. Day 1 followed by 300 mg Q2W.

Dupilumab has been used in an open‐label study for up to 3 years in adults with moderate‐to‐severe AE, but some former trial patients have continued open‐label on the medication for much longer. Safety data were consistent with previously reported trials and the known dupilumab safety profile.^55^

#### Safety

Dupilumab treatment is in general well tolerated, and routine blood tests are not recommended, but a substantial number of patients develop ocular surface disease (over 30% in some ‘real world’ settings), of which most are mild‐to‐moderate.^56^, ^57^, ^58^, ^59^ Topical treatment with anti‐inflammatory eyedrops is often sufficient, without the need to discontinue treatment.^60^

#### Monitoring

No biochemicals or instrumental exams are reported to be required for the monitoring of the therapy.

#### Combination with other treatments

An additional phase III trial evaluated dupilumab treatment and a concomitant TCS compared with placebo and a concomitant TCS over 52 weeks.^61^ The co‐primary endpoints included an IGA score of 0 or 1 and EASI‐75, which were assessed at Week 16: more patients who received dupilumab plus topical corticosteroids achieved the co‐primary endpoints of IGA 0/1 and EASI 75. Results at 52 weeks were similar. Approximately 15% more subjects achieved a 75% reduction in the EASI score at week 16 in this trial compared with previous phase III studies where dupilumab was administered as monotherapy.^53^

Combination therapy with TCS, TCI and UV light treatment is well established.

#### Special considerations

AE patients with type 2 comorbidities such as prurigo nodularis, asthma, chronic rhinosinusitis with nasal polyps (CRSwNP), and/or eosinophilic esophagitis may also have beneficial effects of dupilumab treatment on these diseases.

### Lebrikizumab

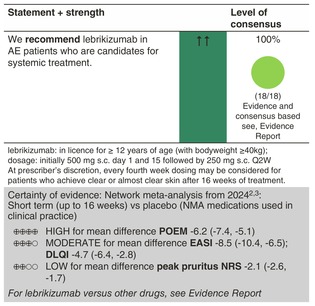

#### Mechanisms of action and efficacy

Lebrikizumab is a high‐affinity humanized immunoglobulin G4 mAb that binds specifically to soluble interleukin 13 and selectively prevents the formation of the IL‐13Rα1/IL‐4Rα heterodimer receptor signalling complex.

In two identically designed randomized, double‐blind, placebo‐controlled monotherapy phase 3 studies (ADvocate1 and ADvocate2), adolescents (aged 12 years and older) and adult AE patients received either lebrikizumab at a dose of 250 mg Q2W (loading dose of 500 mg at baseline and Week 2) or placebo.^62^ After 16 weeks, 43.1% (lebrikizumab arm) versus 12.7% (placebo arm) in ADvocate1 and 33.2% versus 10.8% in Advocate2 achieved the primary endpoint of IGA score of 0 or 1 with a reduction of at least 2 points.^62^ Patients who responded to lebrikizumab at the end of the 16‐week induction period were re‐randomized 2:2:1 to receive lebrikizumab 250 mg Q2W, Q4W or placebo (lebrikizumab withdrawal) for 36 additional weeks. After 52 weeks, an IGA of 0 or 1 with a ≥2‐point improvement was maintained by 71.2% and 76.9% of patients treated with lebrikizumab Q2W or Q4W, respectively. In the lebrikizumab withdrawal arm, only 47.9% maintained their response.^63^ In a further placebo‐controlled phase 3 study, in which the effect of lebrikizumab was investigated together with topical corticosteroids, the drug also proved to be clearly superior to placebo.^64^

#### Dosage: Acute flare, short term, long term

The recommended dosage is an induction therapy of 500 mg at Week 0 and Week 2, followed by 250 mg Q2W until Week 16. In patients with an initial partial response, 250 mg Q2W can be continued until Week 24. Once a clinical response is achieved, the recommended maintenance dose of lebrikizumab is 250 mg Q4W.

#### Safety

Stein Gold et al. investigated the safety of lebrikizumab by analysing data from eight phase 2 and phase 3 studies. Overall, the frequency of adverse events was comparable between patients receiving lebrikizumab and those given a placebo. Conjunctivitis and eczema were the most frequently reported events, with eczema reported more frequently in the placebo groups than in the Q2W groups (18.4% vs. 6.0%) and conjunctivitis reported more frequently in lebrikizumab in the Q2W groups than in the placebo groups (6.5% vs. 1.8%). Nasopharyngitis, headache, allergic conjunctivitis, dry eye and allergic rhinitis were reported more frequently in the lebrikizumab group.^65^

Simpson et al.^66^ reported injection site reactions (1.3%), herpes infection (3.8%), eosinophilia (3.2%) with no associated clinical symptoms and conjunctivitis (9.6%) as adverse events in patients treated with lebrikizumab.

Notably, based on the currently available clinical trial data lebrikizumab appears to have lower rates of ocular complications than dupilumab. 'Real world' comparisons, for instance through national registers, are still awaited.

#### Monitoring

No biochemical or instrumental exams are reported to be required for the monitoring of the therapy.

#### Combination with other treatments

TCS and TCI can be used in conjunction with lebrikizumab, depending on disease and flare control needs.^64^

### Nemolizumab

At the time of the voting process for this guideline update, nemolizumab was not licensed for any indication in the European Union. Therefore, there is currently no specific recommendation for its use in AE in this version of the guideline.

#### Mechanisms of action and efficacy

Nemolizumab is a humanized mAb targeting the IL‐31 receptor alpha chain (IL‐31RA), which was initially developed for the treatment of AE‐related pruritus. The drug is currently also approved for this treatment in Japan.

In two randomized, double‐blind, placebo‐controlled phase 3 trials (ARCADIA 1 and 2), a total of 1728 adults and adolescents (aged ≥12 years) with moderate‐to‐severe AE and associated pruritus were enrolled. All participants had previously shown an inadequate response to TCS treatment. Patients received either 30 mg nemolizumab Q4W, with an initial loading dose of 60 mg, or placebo injections, alongside TCS and, in some cases, TCI. Randomization was conducted in a 2:1 ratio for nemolizumab versus placebo. At Week 16, 44% (270/620) and 42% (220/522) of patients treated with nemolizumab achieved an EASI‐75 response compared to 29% (93/321) and 30% (80/265) of patients receiving placebo. Similarly, the proportion of patients achieving IGA success (defined as an IGA score of 0 or 1 with a reduction of at least 2 points) was significantly higher in the nemolizumab groups than in the placebo groups. Significant benefits were also observed for secondary endpoints, with consistent improvements in pruritus and sleep disturbance.^67^

Patients who achieved a clinical response (IGA success or EASI‐75) at week 16 were re‐randomized (1:1:1) to receive nemolizumab 30 mg Q4W, nemolizumab 30 mg Q8W or placebo for an additional 32 weeks. During this period, additional topical anti‐inflammatory therapy was also permitted. At Week 48, a higher proportion of patients maintained IGA success in the nemolizumab Q4W (61.5%) and Q8W (60.4%) groups compared to the placebo group (49.7%). Similarly, EASI‐75 response rates were higher in the nemolizumab Q4W (76.3%) and Q8W (75.7%) groups compared to placebo (63.9%).^68^

#### Dosage: acute flare, short term, long term

Global phase III trials investigated the nemolizumab 30 mg Q4W dose administered up to Week 16 with a loading dose of 60 mg at baseline and showed positive outcomes. After Week 16, clinical responders did not show a clinical difference with the Q4W and Q8W maintenance regimen.^67^

#### Safety

In the two mentioned phase 3 trials, the frequency of adverse events was comparable between patients receiving nemolizumab and those given a placebo.^67^

The most common treatment‐emergent adverse event was worsening of AE in 12% (75/616) participants in the nemolizumab group versus 11% (34/321) in the placebo group in ARCADIA 1; and 7% (37/519) versus 6% (15/263), respectively, in ARCADIA 2.

Asthma events occurred in both groups in both studies. However, it should be noted that more than 30% of all included patients reported pre‐existing asthma.

In both studies, hypersensitivity reactions (urticaria) were noted. Non‐serious and mild urticaria was reported in 1% and 2% of patients receiving nemolizumab versus <1% of patients receiving placebo in ARCADIA 1 and ARCADIA 2. Nemolizumab was not discontinued due to any of these events.^67^

#### Monitoring

No biochemical or instrumental examinations are reported to be required for the monitoring of the therapy.

#### Combination with other treatments

In phase 3 studies, nemolizumab was investigated with additional treatment with emollients, TCS or TCI therapy.

### Tralokinumab

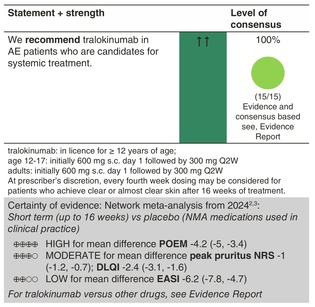

#### Mechanisms of action and efficacy

Tralokinumab is a fully human, high‐affinity IgG4 mAb, which neutralizes IL‐13 and has been approved by the EMA in summer 2021.^69^ In two 52‐week, double‐blind, placebo‐controlled, phase III trials, adults with moderate‐to‐severe AE were randomized to subcutaneous tralokinumab 300 mg every 2 weeks or placebo.^70^ Tralokinumab monotherapy was superior to placebo at 16 weeks of treatment. Co‐primary endpoints were IGA score of 0 or 1 and EASI 75 at week 16. Patients achieving an IGA score of 0/1 and/or EASI 75 with tralokinumab at eek 16 were re‐randomized to tralokinumab Q2W or every 4 weeks or placebo for 36 weeks. The majority of Week 16 tralokinumab responders maintained response at Week 52 with continued tralokinumab treatment without any rescue medication. In a randomized, double‐blind, phase 3 trial, 301 adolescent patients received either 300 or 150 mg of tralokinumab or placebo. After 16 weeks, significantly more patients in the tralokinumab arms showed an EASI 75 response (27.8%, 28.6%, 6.4%) or an IGA of 0 or 1 (17.5%, 21.4%, 4.3%). Subjects achieving a clinical response (IGA = 0, 1; or EASI75) at Week 16 without use of rescue medication were re‐randomized to maintenance dosing regimens. At 52 weeks, EASI‐75 response ranged from 44.4% to 63.3% in the different maintenance dosing regimens.^71^

#### Dosage: acute flare, short term, long term

The recommended dosage is 300 mg every 2 weeks after a loading dose of 600 mg at treatment onset. At the prescriber's discretion, every fourth week dosing may be considered for patients who achieve clear or almost clear skin after 16 weeks of treatment.

Phase III trials have also investigated what happens when patients who do well for 16 weeks on tralokinumab continue treatment as labelled, reduce treatment frequency or discontinue treatment.

After 16 weeks, patients who reached EASI 75 or IGA success were re‐randomized to continue treatment every 2 weeks, titrate down to every 4 weeks or use placebo. At 52 weeks, without TCS, more than 55% of patients who continued twice‐monthly treatment maintained EASI 75, as did approximately 50% of patients treated monthly. More than 51% of patients who stayed on twice‐monthly dosing maintained IGA 0 or 1, versus 39% and 45% of patients who switched to monthly dosing.

#### Safety

In the two studies, adverse events were reported in 76.4% and 61.5% of patients receiving tralokinumab and in 77.0% and 66.0% of patients receiving placebo in the 16‐week initial period.

Notably, based on the currently available clinical trial data, tralokinumab appears to have lower rates of ocular complications than dupilumab.^70^

The combination therapy with TCS, TCI and UV light treatment is possible.

#### Monitoring

No biochemical or instrumental exams are reported to be required for the monitoring of the therapy.

#### Combination with other treatments

In an additional phase III double‐blind, placebo study, the efficacy and safety of tralokinumab in combination with TCS as needed in patients with moderate‐to‐severe AE were evaluated. At Week 16, significantly more tralokinumab‐treated patients than placebo achieved IGA 0/1 and EASI 75. Nine out of ten EASI 75 responders at Week 16 maintained response at Week 32 with continued tralokinumab and TCS as needed.^72^

### Omalizumab

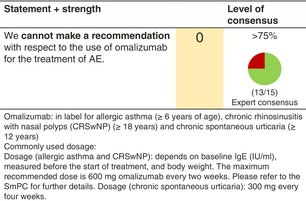

#### Mechanisms of action and efficacy

Most AE patients have elevated serum IgE levels, but the pathogenic role of IgE in AE remains unknown. The anti‐IgE antibody omalizumab has been used with great success for the treatment of chronic spontaneous urticaria (CSU). A recent systematic review and meta‐analysis has assessed the preclinical and trial data regarding omalizumab treatment of AE, which are conflicting.^73^

Omalizumab is licensed for the treatment of asthma and CSU, but not for the treatment of AE.

Omalizumab is binding free IgE, which leads to immune complexes of IgE and omalizumab. IgE bound to omalizumab cannot bind to the alpha chain of the high‐affinity receptor for IgE, thereby inhibiting its binding to mast cells, basophils and epidermal dendritic cells^74^, ^75^ and subsequent immunological effects.

There are many case reports and case series,^73^ but only a few controlled trials studying omalizumab treatment of AE.^73^, ^76^ In summary, the data show a measurable but moderate efficacy of omalizumab for improving signs and symptoms of AE.^73^, ^77^ There is no predictive marker linked to a better clinical response, and most of the published evidence is of low quality. The safety of omalizumab is very good,^73^ but the unpredictable and statistically low efficacy prevents a general recommendation for omalizumab regarding the treatment of AE.

#### Dosage: acute flare, short term, long term

##### Adult

Different dosages have been tested in AE patients, ranging from 150 to 450 mg every 2 weeks or every 4 weeks. A recent systematic review and meta‐analysis by Wollenberg et al.^73^ found that patients with lower baseline IgE showed a positive response to treatment with omalizumab compared with patients with very high to extremely high serum IgE.

An older systematic review and meta‐analysis by Wang et al.^78^ also found that IgE serum concentrations of lower than 700 IU/mL were associated with a better clinical response, compared with IgE concentrations of 700 to >5000 IU/mL. Age, sex, baseline clinical disease severity, the history of concomitant asthma and the use of 600 mg/month or more of omalizumab showed no significant association with the clinical results associated with omalizumab use.

##### Children

The ADAPT (Atopic Dermatitis Anti‐IgE Paediatric Trial) trial evaluated the possible role of omalizumab in the management of severe paediatric AE with concomitant allergic disease (asthma, allergic rhinoconjuncitivitis or food allergies) for 24 weeks. The drug dose was determined by baseline total IgE (range: 30 to 1500 IU/mL), measured before the start of treatment, and body weight (kg) and calculated using the formula: 0.016 × weight (kg) × total IgE level (kU/L) in 2–4 weekly injections. The study showed that omalizumab significantly reduced disease severity and improved QoL in paediatric patients with severe AE and highly elevated IgE levels (median baseline total IgE of 8373 IU/L) compared with placebo.^76^ However, this improvement was below the minimal clinically important difference for the main outcome (objective SCORAD).

#### Safety

There is a general consensus about the overall good safety profile of omalizumab, with some controlled studies reporting excellent tolerability up to 4 years. A 2009 revision of data from controlled trials concluded that the incidence of anaphylaxis was 0.14% in omalizumab‐treated patients and 0.07% in control subjects. Of note, no serum sickness attributable to the drug and no anti‐omalizumab antibodies have been reported to date.^79^

There are no reported interactions of omalizumab with other medications used for AE or other allergic diseases. If clinically needed, omalizumab may be considered during pregnancy. More attention has been put on the appearance of gut parasite infections in treated patients, since IgE is an important player in the host defence against parasitic helminths. A randomized placebo‐controlled trial in 137 adult subjects with respiratory allergy at high risk of helminth infection showed a modest increase in the incidence of parasitism in the active group.^80^

#### Monitoring

No biochemicals or instrumental exams are reported to be required for the monitoring of the therapy. IgE levels increase following the administration of omalizumab and may remain elevated for up to 1 year following the discontinuation of the drug.

## JAK‐INHIBITORS

The Janus kinase (JAK) family, constituting JAK1, JAK2, JAK3 and tyrosine kinase 2 (TYK2), is a class of cytoplasmic tyrosine kinases.^81^ JAKs dock to the intracellular part of cytokine receptor chains to generate functional signalling complexes and regulate the inflammatory process through activating the intracytoplasmic transcription factors termed as signal transducer and activator of transcription (STAT). When activated, STAT proteins produce dimers, which translocate into the nucleus and either positively or negatively regulate downstream target gene expression of inflammatory mediators, suggesting that inhibiting JAK activity may be more effective than targeting a single cytokine. Past the disruption of cutaneous inflammatory cytokine signalling, JAK inhibition has been reported to attenuate chronic itch and improve skin barrier function by regulating the expression of the skin barrier protein filaggrin.^82^, ^83^

### Special considerations

AE patients with concomitant inflammatory diseases, such as alopecia areata, rheumatoid and juvenile idiopathic arthritis, ankylosing spondylitis, psoriatic arthritis and inflammatory bowel diseases are likely to experience beneficial effects, but the effect size may vary according to the JAK inhibitor used.

### General safety issues

Following a review of the benefit–risk balance of oral JAK inhibitors, which confirmed that tofacitinib (oral JAK inhibitor; not approved for AE) increases the risk of major cardiovascular problems, cancer, venous thromboembolism and serious infections compared to TNF‐alpha inhibitors,^84^ the Pharmacovigilance Risk Assessment Committee (PRAC) of the European Medicines Agency (EMA) recommended measures to minimize the risk of serious side effects associated with JAK inhibitors in 2022.^85^, ^86^ It was recommended that oral JAK inhibitors should only be used in patients aged ≥65 years or with a history of atherosclerotic cardiovascular disease, other risk factors for cardiovascular disease (e.g. long‐term smoking) or with an increased risk of cancer if no suitable treatment alternatives are available, and with caution in patients at risk of pulmonary embolism or deep vein thrombosis.^85^, ^86^ The PRAC concluded that these safety findings apply to all approved uses of JAK inhibitors in dermatological, rheumatological or gastroenterological chronic inflammatory diseases.^85^, ^86^

### Monitoring

Per JAK inhibitor, the label's requirements for monitoring are mentioned. In practice, we recommend the same baseline screening and treatment monitoring investigations for all JAK inhibitors. For baseline screening, this is a full blood count, renal, liver and lipid profile as well as creatinine phosphokinase levels and hepatitis, HIV and TB screen. For monitoring purposes during treatment, we recommend a full blood count, renal, liver and lipid profile as well as creatinine phosphokinase level at 4 weeks into treatment and then 3‐monthly while on therapy.

### Pregnancy

All JAK inhibitors are considered strictly contraindicated during pregnancy.

### Abrocitinib

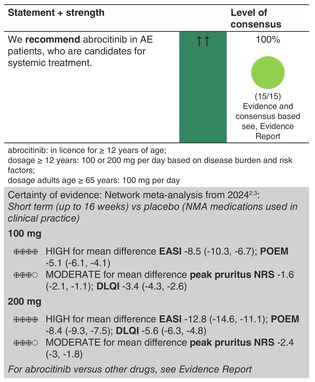

#### Mechanisms of action and efficacy

Abrocitinib is an oral JAK1 selective inhibitor and has shown efficacy in patients with moderate‐to‐severe AE when used as a monotherapy (MONO‐1 and ‐2 studies) and in combination with topical therapies in achieving treatment response in comparison to placebo (COMPARE study), as measured using IGA and EASI‐75 response. For instance, the proportion of patients with EASI‐75 response at week 12 was significantly higher with abrocitinib 100 mg (~40%–45%) and abrocitinib 200 mg (~61%–63%) compared to placebo (~10%–12%) in the MONO studies. In the COMPARE study, the proportion of patients with EASI‐75 response was significantly higher with abrocitinib 100 mg (~59%) and abrocitinib 200 mg (~70%) compared to placebo (27%).^11^ Similar efficacy has been demonstrated in the adolescent JADE TEEN trial for both the 100 mg and 200 mg doses, in combination with topical therapies.^87^ Importantly, in the COMPARE study (which had dupilumab as a comparator arm) higher responder rates were observed with abrocitinib 200 mg compared to dupilumab (*p*‐values not calculated) after 16 weeks of treatment. The efficacy of abrocitinib 100 mg and dupilumab was similar in this subgroup. The results indicate that abrocitinib 200 mg may provide a higher probability of treatment response compared to dupilumab in patients with severe AE.^88^

#### Dosage: acute flare, short term, long term

Abrocitinib is licensed at the 100 mg and the 200 mg daily doses, with the lower dose recommended for adolescents as a starting dose. One study assessed flare prevention, that is, the risk and probability of flares and recapture of treatment response following a flare. Of 1233 patients, 798 responders to induction with abrocitinib 200 mg (64.7%) were randomly assigned to dose maintenance, dose reduction or treatment withdrawal (placebo). The flare probability during maintenance was 18.9%, 42.6% and 80.9% with abrocitinib 200 mg, abrocitinib 100 mg and placebo, respectively, by Week 52. Among patients with flare in the abrocitinib 200 mg, abrocitinib 100 mg and placebo groups, 36.6%, 58.8% and 81.6% regained IGA 0/1 response, respectively, and 55.0%, 74.5% and 91.8% regained EASI index response, respectively, with rescue treatment of abrocitinib 200 mg plus medicated topical therapy.^89^

#### Safety

Based on long‐term follow‐up of patients from the phase II and III trials as well as one long‐term extension study, with a total *n* of 2856 (1614 patient‐years (PY); total exposure in the all‐abrocitinib cohort was ≥24 weeks in 1248 patients and ≥48 weeks in 606 (maximum 108 weeks)). In the placebo‐controlled cohort (*n* = 1540), dose‐related adverse events (200 mg, 100 mg, placebo) were nausea (14.6%, 6.1%, 2.0%), headache (7.8%, 5.9%, 3.5%) and acne (4.7%, 1.6%, 0%). Platelet count was reduced transiently in a dose‐dependent manner; 2/2718 patients (200‐mg group) had confirmed platelet counts of <50 × 10^3^/mm^3^ at Week 4. Incidence rates (IRs) were 2.33/100PY and 2.65/100PY for serious infection, 4.34/100PY and 2.04/100PY for herpes zoster and 11.83/100PY and 8.73/100PY for herpes simplex in the 200‐ and 100‐mg groups, respectively.^90^

#### Monitoring

For baseline screening, the manufacturer's UK label laboratory monitoring recommendations are full blood count including platelet count, absolute lymphocyte count (ALC), absolute neutrophil count (ANC) and haemoglobin (Hb) as well as lipid parameters. Creatinine phosphokinase level and an infection screening for HIV, hepatitis B and C, as well as TB are advisable before initiation of therapy.

In practice, we recommend the same baseline screening and treatment monitoring investigations for all JAK inhibitors. For baseline screening, this is a full blood count, renal, liver and lipid profile as well as creatinine phosphokinase levels and hepatitis, HIV and TB screen.

For monitoring purposes, we recommend a full blood count, renal, liver and lipid profile as well as creatinine phosphokinase level at 4 weeks into treatment and then three‐monthly while on therapy.

#### Combination with other treatments

No studies assessing the use of abrocitinib with other systemic therapies have been published to date.

#### Special considerations

Abrocitinib is a new JAK inhibitor and has not been formally tested in other inflammatory diseases.

Please refer to the special considerations in the JAK inhibitor introduction section.

### Baricitinib

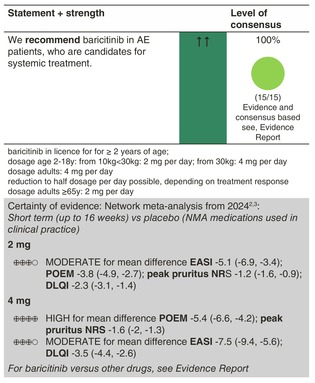

#### Mechanisms of action and efficacy

Baricitinib is an oral selective JAK1 and JAK2 inhibitor. The drug has been tested in one phase 2 and several phase 3 trials in adults with moderate‐to‐severe AE at 1, 2 and 4 mg once daily against placebo, showing significant improvement with regard to EASI from baseline to 16 weeks, in particular in the two higher doses (2 mg daily (mean difference, 5.1‐point reduction; 95% CI, −6.9, −3.4 [GRADE assessment: moderate certainty]) and 4 mg daily (mean difference, 7.5‐point reduction; 95% CI, −9.4, −5.6 [GRADE assessment: moderate certainty])).^2^ Similar efficacy has been shown in these studies with regard to the IGA and itch scores. The concomitant use of topical corticosteroids was allowed in one trial.^91^

In October 2023, baricitinib was approved by the EMA for the treatment of children (2 years and older) and adolescents with AE. Data from a phase 3 study (BREEZE‐AD PEDS) had previously been published, in which 483 children and adolescents received baricitinib 0.5 mg or 1 mg or 2 mg or 4 mg or placebo over 16 weeks. The drug was convincing with regard to the primary endpoint Investigator Global Assessment of 0/1 with a ≥2‐point improvement at Week 16 as well as the secondary endpoints EASI‐75, EASI‐90, mean change from baseline in EASI score, SCORAD 75 and 4‐point improvement in the Itch Numeric Rating Scale.^92^

#### Dosage: acute flare, short term, long term

At present, baricitinib data are available up to 52 weeks follow‐up,^93^ demonstrating sustained efficacy.

The recommended dose of baricitinib for adult patients is 4 mg once daily. A dose of 2 mg once daily should be considered for patients who have achieved sustained control of disease activity with 4 mg once daily and are eligible for dose tapering.

A dose of 2 mg once daily is recommended for patients at higher risk of venous thromboembolism, major adverse cardiovascular events and malignancy, as well as for patients aged ≥65 years and for patients with a history of chronic or recurrent infections. A dose of 4 mg once daily may be considered for these patients who do not achieve adequate control of disease activity with a 2‐mg once daily dose.

For children and adolescents, the recommended dose of baricitinib is 4 mg once daily for patients weighing 30 kg or more. For patients weighing 10–30 kg, the recommended dose is 2 mg once daily. A reduction to half the dose should be considered for patients who have achieved sustained control of disease activity with the standard recommended dose and are eligible for dose tapering.

#### Safety

The most common side effects with baricitinib in clinical trials include an increase in LDL cholesterol, upper respiratory tract infections and headache. Acne is less common than with other JAK inhibitors. Infections reported with baricitinib include herpes simplex. However, the rate of these events reported in a recent combined safety study including 2531 patients with AE from eight RCTs who were given baricitinib for 2247 patient‐years (median duration 310 days) was overall low: eczema herpeticum (*n* = 11), cellulitis (*n* = 6) and pneumonia (*n* = 3). There were four opportunistic infections reported.^94^ A transient increase of CPK may be seen, especially after extensive bodily exercise. No malignancies, gastrointestinal perforations, positively adjudicated cardiovascular events or tuberculosis were reported in the placebo‐controlled period in baricitinib‐treated patients. The frequency of herpes simplex was higher in the 4 mg group (6.1%) compared to the 2 mg (3.6%) and placebo groups (2.7%). Analyses of long‐term safety data are still pending in patients with AE. For use in rheumatoid arthritis, data is available from an integrated database with 9 phase 3,2,1b trials and 1 long‐term extension covering 3,770 patients for up to 9.3 years of baricitinib treatment (14,744 patient‐years of exposure).^95^ Patients who received at least one dose of baricitinib in the incorporated studies were included in the uncontrolled analysis set. The incidence rate for serious infections was reported with 2.6 per 100 patient years, while the incidence rate for herpes zoster was reported with 3 per 100 patient years. The incidence rate for major cardiovascular events was 0.5 per 100 patient years, rising slightly to 0.77 in those patients over 50 with at least one cardiovascular risk factor. Incidence rates for deep vein thrombosis or pulmonary embolism were reported with 0.5 per 100 patient years.^96^

In the children and adolescents BREEZE‐AD PEDS study, abdominal pain, acne and headache were the most frequently reported adverse events. Few patients discontinued the study drug due to AEs (1.6% placebo and 0.6% baricitinib‐treated).^92^

#### Monitoring

For baseline screening, the manufacturer advises that patients with suspected hepatitis B consult a liver specialist for advice before the initiation of treatment. Lipid and liver profiles need to be regularly monitored following treatment initiation. Screening for any haematological abnormalities is also advised.

In practice, we recommend the same baseline screening and treatment monitoring investigations for all JAK inhibitors. For baseline screening, this is a full blood count, renal, liver and lipid profile as well as creatinine phosphokinase levels and hepatitis, HIV and TB screen.

For monitoring purposes, we recommend a full blood count, renal, liver and lipid profile as well as creatinine phosphokinase level at 4 weeks into treatment and then 3‐monthly while on therapy.

#### Combination with other treatments

No studies assessing the use of baricitinib with other systemic therapies in AE patients have been published to date, but the combination therapy with MTX is an established combination regimen in the management of rheumatoid arthritis.^97^

#### Special considerations

JAK inhibitors are also effective for certain other inflammatory diseases and are partially approved for their treatment. Therafore,  patients with AE and with concomitant inflammatory diseases, such as AA, rheumatoid and juvenile idiopathic arthritis, ankylosing spondylitis and psoriatic arthritis and inflammatory bowel diseases are likely to experience additional beneficial effects for these concomitant diseases. Baricitinib is already licensed for most of these indications.

Please refer to the special considerations in the JAK inhibitor introduction section.

In 2023, an analysis was published that evaluated pooled safety data from randomized clinical trials and long‐term extensions of patients treated with baricitinib for rheumatoid arthritis (RA), AE or AA.^98^ The patients were divided into a low‐risk group (younger than 65 years with no specified risk factors) and a risk group (≥1 of: aged 65 years or older, atherosclerotic cardiovascular disease, diabetes mellitus, hypertension, current smoking, HDL cholesterol <40 mg/dL, BMI ≥30 kg/m^2^, poor mobility on EQ‐5D or history of malignancy). In the low‐risk group, the incidence rate (IR) per 100 patient‐years was for major adverse cardiovascular event (MACE) (0.05, 0.04, 0), malignancies (0.20, 0.13, 0), VTE (0.09, 0.04, 0), serious infection (1.73, 1.18, 0.6) and mortality (0.04, 0, 0) in the RA, AD, and AA datasets, respectively. In patients at risk, IRs were for MACE (0.70, 0.25, 0.10), malignancies (1.23, 0.45, 0.31), VTE (0.66, 0.12, 0.10), serious infection (2.95, 2.30, 1.05) and mortality (0.78, 0.16, 0) for RA, AD and AA datasets, respectively.^98^ However, it is important to compare such risk estimates against the background population risk, as clinical trials are short‐term and many patients with significant comorbidities are not eligible for AE trial enrolment. The same applies to other JAK inhibitors as well.

### Upadacitinib

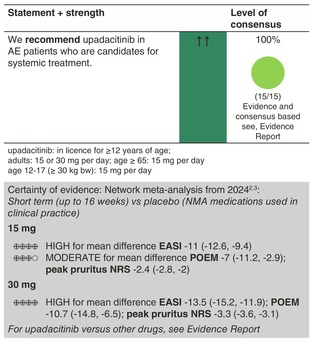

#### Mechanisms of action and efficacy

Upadacitinib is a relatively selective and reversible Janus kinase (JAK) 1 inhibitor. There is one phase 2 trial including 167 adult patients that investigated three different doses of upadacitinib (30, 15 and 7.5 mg/day) for AE compared to placebo.^99^ The trial was conducted over 16 weeks. Upadacitinib was superior to placebo for all dosage groups in EASI (mean change (SE) 74% (6.1%) for 30 mg, 62% (6.1%) for 15 mg, 39% (6.2%) for 7.5 mg and 23% (6.4%) for placebo (*p* = 0.03, <0.001, <0.001)). There were also significant improvements seen with regard to the SCORAD index, NRS pruritus and POEM scores. The phase 3 trials published since have shown similar efficacy.^100^, ^101^, ^102^

In a direct head‐to‐head trial enrolling adult AE patients randomized to receive upadacitinib (*n* = 348) and dupilumab (*n* = 344), 247 patients receiving upadacitinib (71.0%) and 210 patients receiving dupilumab (61.1%) achieved EASI75 at 16 weeks (*p* = 0.006). All ranked secondary endpoints also demonstrated the superiority of upadacitinib versus dupilumab, including improvement in Worst Pruritus NRS as early as Week 1, achievement of EASI75 as early as Week 2 and EASI100 at Week 16. Rates of serious infection, eczema herpeticum, herpes zoster and laboratory‐related adverse events were higher for patients who received upadacitinib, whereas rates of conjunctivitis and injection‐site reactions were higher for patients who received dupilumab.

#### Dosage: acute flare, short term, long term

Upadacitinib is licensed at the 15 and 30 mg doses for AE, and at 15 mg for rheumatoid arthritis, psoriatic arthritis and ankylosing spondylitis. Follow‐up until eek 52 is now available, showing long‐term efficacy and safety profiles similar to the 16‐week trials.^103^ There is no study that has looked at acute flare treatment, and there are currently early phase AE trials in children >6 months.

#### Safety

The cumulative incidence rates of adverse events were 78.6% for 30 mg, 76.2% for 15 mg, 73.8% for 7.5 mg and 62.5% for placebo in the phpase 2 trial and have been similar in the studies reported since.^99^ Upper respiratory tract infections and acne were the most frequently reported adverse events for upadacitinib. The cumulative incidence rates of severe adverse events were 0% for 30 mg, 2.4% for 15 mg, 4.8% for 7.5 mg and 2.4% for placebo. Low withdrawal rates were reported in the placebo and upadacitinib groups (*n* < 5 for each group). In a phase 3 trial, 272 Japanese patients (age 12–75 years) with moderate‐to‐severe AE were randomized in a 1:1:1 ratio to receive 15 mg upadacitinib, 30 mg upadacitinib or placebo (each in combination with a TCS) to evaluate the safety of upadacitinib in combination with TCS. Treatment‐emergent adverse events (TEAEs) were reported for 56.0%, 63.7% and 42.2% of participants, respectively, at Week 24. The most frequently reported TEAEs were acne (13.2%, 19.8%, 5.6%), nasopharyngitis (13.2%, 15.4%, 15.6%) and herpes zoster infection (0%, 4.4%, 0%). No thromboembolic events, malignancies, gastrointestinal perforations or deaths occurred.^104^

#### Monitoring

The manufacturer advises that patients are screened for viral hepatitis B and C and TB. Lipid and liver profiles need to be measured at baseline and regularly following treatment initiation. Screening and monitoring for any haematological abnormalities are also advised, no later than 12 weeks.

In practice, we recommend the same baseline screening and treatment monitoring investigations for all JAK inhibitors. For baseline screening, this is a full blood count, renal, liver and lipid profile as well as creatinine phosphokinase levels and hepatitis, HIV and TB screen.

For monitoring purposes, we recommend a full blood count, renal, liver and lipid profile as well as creatinine phosphokinase level at 4 weeks into treatment and then 3‐monthly while on therapy.

#### Combination with other treatments

No studies assessing the use of upadacitinib with other systemic therapies in AE patients have been published to date, but the combination therapy with MTX is an established combination regimen in the management of rheumatoid arthritis, albeit only with the 15 mg once a day dose.^105^

#### Special considerations

JAK inhibitors are also effective for certain other inflammatory diseases and are partially approved for their treatment. Therefore, patients with AE and with concomitant inflammatory diseases, such as AA, rheumatoid and juvenile idiopathic arthritis, ankylosing spondylitis, psoriatic arthritis and inflammatory bowel diseases, are likely to experience additional beneficial effects for these concomitant diseases. Upadacitinib is already licensed for most of these indications.

Please refer to the special considerations in the JAK inhibitor introduction section.

## PREGNANCY, BREASTFEEDING AND FAMILY PLANNING

The current ethical framework of GCP guidelines deems it unethical to perform clinical trials in pregnant women. Therefore, there is no high‐level evidence data on the efficacy and safety in this patient population. On the other hand, AE is the most common general skin disease in pregnancy. AE may either (i) worsen in women with a chronic condition, or (ii) be reactivated in patients with a past AE history or (iii) occur in women with no AE history (atopic eruption of pregnancy, AEP). Worsening of AE is mostly reported during the second and third trimesters, while AEP typically occurs during the first trimester.^20^ There are no major clinical differences between classical AE worsening and AEP. Physiological skewness of the immune system towards a Th2‐dominated response during pregnancy as well as physical and psychological stress during this period may contribute to AE worsening during pregnancy. Little is known about treatment patterns during pregnancy, but patients and caregivers tend to reduce the use of topical and systemic therapies during pregnancy to avoid presumed harm to the fetus.^106^ Consequently, undertreatment of AE during pregnancy may lead to serious QoL impairment but also to complications such as eczema herpeticum or staphylococcus aureus skin infections, and should therefore be avoided.

### Pregnant women

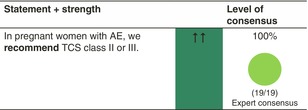

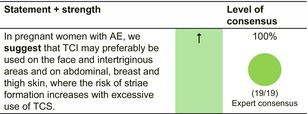

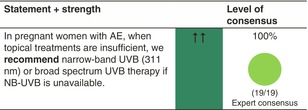

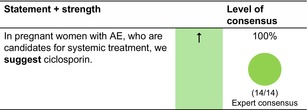

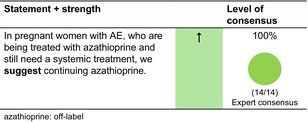

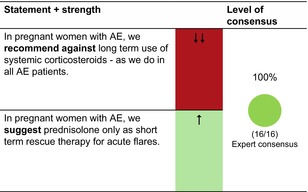

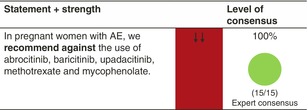

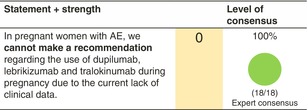

#### First‐line treatments

##### Emollients

Basic emollient therapy is key in the treatment of AE also during pregnancy and must be proposed to pregnant women with AE as a basic daily therapy. There is no firm evidence on which emollient should be used, but using one with a high‐lipid content and as few potentially harmful agents as possible is recommended. Using emollients in a wet wrap technique is encouraged.^107^

##### TCS

Reactive or proactive use of TCS class II or III is recommended. A Cochrane systematic review updated in 2015, including 14 studies (5 cohort and 9 case–control studies) with 1,601,515 study subjects, has examined the risk of TCS use in pregnancy. Overall, it has been deemed safe, with no causal associations between maternal exposure to TCS of all potencies and pregnancy outcomes, including mode of delivery, congenital abnormalities, preterm delivery, fetal death and low Apgar score, although the use of very potent topical corticosteroids may be associated with low birthweight.^108^ Proactive, twice‐weekly TCS application as maintenance therapy is regarded as safe, but caution is recommended when using potent TCS over large body surface areas or sensitive areas such as breast and thigh skin on a more regular basis. Some experts suggest that Class IV may be used as rescue therapy or over longer periods on limited skin areas, but this is controversial. Fluticasone propionate should be avoided as it is the only TCS that is known not to be metabolized by the placenta.^20^ There is experimental data that there is zero degradation of fluticasone propionate at the placental barrier, whereas the degradation of hydrocortisone is high and the degradation of betamethasone is low.^109^, ^110^ Therefore, we specifically recommend not to use fluticasone propionate in pregnant women.^20^

##### TCI

Reactive and proactive use of TCI may be preferable on the face and intertriginous areas, and on abdominal, breast and thigh skin, where the risk of striae formation increases with excessive use of TCS.

##### Antiseptics

Antiseptics, except triclosan, may be used by pregnant women if clinically needed to prevent recurring skin infections but are not recommended as a general routine measure.

##### UV phototherapy

Therapy with narrow‐band UVB (311 nm) and broad‐spectrum UVB does not impose a risk to the fetus in pregnant women. However, oral psoralen should not be used preconceptionally (3 months) or in pregnant women.

#### Second‐ and third‐line treatments

Second‐ and third‐line treatments are recommended for pregnant women with AE who are inadequately controlled with TCS class II or III.

Systemic corticosteroids should not be used in the long term in AE in general and even more so not during pregnancy, as it is associated with an increased risk of fetal complications, including gestational diabetes.^108^ Only short courses of prednisolone (maximum 0.5 mg/kg/day) may be used with a strict indication.

Ciclosporin may be used off‐label in severe uncontrolled AE during pregnancy if topical anti‐inflammatory treatment alone or in combination with UV treatment fails, and there is a clear need for better long‐term disease control. However, extra attention should be given to the renal function and blood pressure of the mother. There is no evidence of teratogenicity. Ciclosporin crosses the placenta^111^ and should not be used during pregnancy unless the potential benefit to the mother justifies the potential risk to the fetus.

AZA may be used off‐label in pregnant women with severe uncontrolled AE, who are already receiving this treatment at the time of conception. There is no evidence for teratogenicity from studies with patients with inflammatory bowel diseases. Closely consulting an experienced obstetrician when prescribing this drug is strongly recommended.^20^

MTX and mycophenolate mofetil are teratogenic and are therefore strictly contra‐indicated during pregnancy.

We cannot recommend any of the novel systemic medications, as there is currently no clinical data available to inform about any potential drug‐associated risks. On the other hand, pre‐clinical data do not indicate that there would be a teratogenic potential of dupilumab, lebrikizumab or tralokinumab if given during pregnancy.

Some physicians have used dupilumab during pregnancy, but experience, evidence and data remain limited.^112^, ^113^, ^114^, ^115^, ^116^, ^117^, ^118^

Abrocitinib, baricitinib and upadacitinib are contraindicated during pregnancy according to the label. There are no clinical data but single case reports supporting their safety in pregnant women; however, teratogenic effects have been described for both molecules in animal models.

Antihistamines are of limited efficacy in AE (see chapter antipruritic treatment). In case of need, loratadine should preferentially be used because of the broad experience with this drug in pregnant women.

Due to a lack of experience with crisaborole during pregnancy, this drug should not be used preconceptionally, in pregnancy or during lactation.

### Specific consideration for breastfeeding women

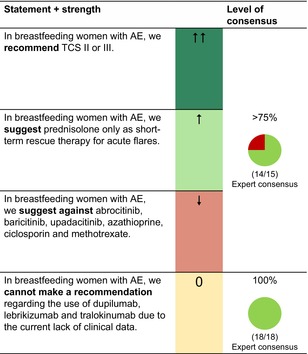

TCS and TCI: No studies have examined the safety of TCS and TCI use during lactation, but no harmful effect is suspected. Nevertheless, it is recommended to apply the topical treatment in the nipple region immediately after nursing the child to allow the drug to be absorbed into the skin before the next feeding.^20^

Systemic corticosteroids: Treatment with a short course of a systemic corticosteroids during lactation is safe, as <0.1% of the mother's ingested dosage is secreted into breastmilk.

With regard to biologics (dupilumab, lebrikizumab, tralokinumab), we are currently unable to make a recommendation for or against their use in breastfeeding mothers due to a lack of clinical data. However, there are indications that the concentration of biologics in general (not just IL4 and IL13 inhibitors) appears to be low in breast milk.^119^

MTX, AZA, ciclosporin and JAK inhibitors are secreted in breastmilk and may induce immunosuppression in the neonate. MTX, AZA, ciclosporin and JAK inhibitors are generally not recommended for lactating mothers.^20^

### Family planning

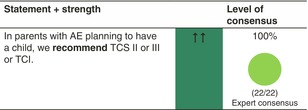

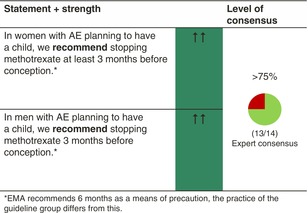

#### Preconception recommendations for women

TCS and TCI: Although the literature on this subject is very sparse, topical AE therapies in women wishing to conceive can be used without concern.

MTX: Local labels in different countries suggest a contraindication range spanning from 1 month to 6 months before conception. The European Medicines Agency (EMA) recommends 6 months as a means of precaution. The practice of the guideline group differs from this, and we recommend stopping methotrexate 3 months before conception.

#### Preconception recommendations for men

TCS and TCI: Although the literature on this subject is very sparse, topical AE therapies in men wishing to father a child can be used without concern.

Ciclosporin may be used in the treatment of AE in men at the time of conception, as there is no evidence of harm or decreased fertility.

MTX: Following the European S3‐guideline on systemic treatment of psoriasis vulgaris, a 3‐month MTX pause prior to conception is recommended. However, (inadvertent) exposure beyond this time does not justify termination of pregnancy because there is no evidence of male teratogenicity.^20^

AZA and JAKi: there is no contraindication for the use of AZA and JAKi in men wishing to father a child.

Dupi, Tralo, Lebri: There is currently no evidence that men need to abstain from fathering a child while on therapy. Further data is awaited to clearly demonstrate evidence of no harm.

## AUTHOR CONTRIBUTIONS

**A. Wollenberg:** Conceptualization, project administration, supervision, visualization, writing – original draft, writing – review and editing. **M. Kinberger:** Methodology, project administration, supervision, visualization, writing – original draft, writing – review and editing. **B. Arents:** Writing – original draft, writing – review and editing. **N. Aszodi:** Writing – original draft, writing – review and editing. **S. Barbarot:** Writing – original draft, writing – review and editing. **T. Bieber:** Writing – original draft, writing – review and editing. **H. A. Brough:** Writing – original draft, writing – review and editing. **P. Calzavara‐Pinton:** Writing – original draft, writing – review and editing. **S. Christen‐Zaech:** Writing – original draft, writing – review and editing. **M. Deleuran:** Writing – original draft, writing – review and editing. **M. Dittmann:** Methodology, software. **N. Fosse:** Writing – original draft, writing – review and editing. **K. Gáspár:** Writing – original draft, writing – review and editing. **L. A. A. Gerbens:** Writing – original draft, writing – review and editing. **U. Gieler:** Writing – original draft, writing – review and editing. **G. Girolomoni:** Writing – original draft, writing – review and editing. **S. Gregoriou:** Writing – original draft, writing – review and editing. **S. Holland:** Writing – original draft, writing – review and editing. **C. G. Mortz:** Writing – original draft, writing – review and editing. **A. Nast:** Conceptualization, methodology, project administration, supervision, visualization, funding acquisition, writing – review and editing. **U. Nygaard:** Writing – original draft, writing – review and editing. **E. M. Rehbinder:** Writing – original draft, writing – review and editing. **J. Ring:** Writing – original draft, writing – review and editing. **M. Rossi:** Writing – original draft, writing – review and editing. **E. Serra‐Baldrich:** Writing – original draft, writing – review and editing. **D. Simon:** Writing – original draft, writing – review and editing. **Z. Z. Szalai:** Writing – original draft, writing – review and editing. **J. C. Szepietowski:** Writing – original draft, writing – review and editing. **A. Torrelo:** Writing – original draft, writing – review and editing. **T. Werfel:** Writing – original draft, writing – review and editing. **R. N. Werner:** Conceptualization, methodology, project administration, supervision, visualization, writing – review and editing. **C. Flohr:** Conceptualization, project administration, supervision, visualization, writing – original draft, writing – review and editing.

## FUNDING INFORMATION

This update of the EuroGuiDerm Guideline was funded through the EuroGuiDerm Centre for Guideline Development. The European Dermatology Forum is responsible for fundraising and holds all raised funds in one account. The EuroGuiDerm Team is not involved in fundraising or in the decision‐making on which guideline (GL) or consensus statement (CS) development is funded. The decisions on which GL/CS is funded are made by the EuroGuiDerm Board of Directors independently. The European Dermatology Forum (EDF) or any other body supporting the EuroGuiDerm is never involved in the guideline development and has had no say on the content or focus of the guideline.

## CONFLICT OF INTEREST STATEMENT

This is a summary of the second update of the EuroGuiDerm Guideline on atopic eczema. For the complete guideline, methods report (including COI disclosures) and evidence report, see https://www.guidelines.edf.one/.

## ETHICAL APPROVAL

Not applicable.

## ETHICS STATEMENT

Not applicable.

## Data Availability

Data sharing is not applicable to this article as no new data were created or analysed in this study.
